# Supplementary material for: Assessing acceptance of augmented reality in nursing education
Source: PLoS One. 2022 Feb 17;17(2):e0263937. doi: 10.1371/journal.pone.0263937 (PMC8853491; doi:10.1371/journal.pone.0263937)
Supplement: Attachment — Submitted filename: Response to Reviewers comments.docx [file pone.0263937.s002.docx]

Dear Editor,

We appreciate you and the reviewers for taking the time to read our work and provide important feedback. Your important and informative feedback has resulted in possible changes in the current version. We attentively considered the comments and did our best to address each one. We hope that the manuscript, following rigorous edits, meets your high expectations. If there are any further constructive remarks, the writers would appreciate them.

The point-by-point replies are provided below. All changes have been indicated in green in the document. (**The reviewer’s** comments are in ***italics***)

**Reviewer #1:**

***The authors aimed to investigate the acceptance of AR in nursing education. They used s a quantitative research method by collecting 419 nursing students’ online feedback to the questionnaire items. In addition to examining the linear relationships between using the structural equation modeling, they also used artificial neural networks to determine the non-linear relationships between constructs.***

***The topic is interesting and the study is generally well conducted. However, the background and literature review sections are weak.***

As suggested by the reviewer, we have developed the background and literature section. All changes have been indicated in green on the manuscript. References list increase from 53 to 80.

***The authors need to state in detail why investigating nursing students’ AR acceptance and the use of ANN are needed by referring to the literature.***

As suggested by the reviewer, on page 11, the following section has been added.

One disadvantage of using SEM is the possibility of ignoring the unforeseen relationships in the research model because it analyzes through defined relationships. Therefore, in the second stage, the nonlinear relationships between the constructs were tested with an Artificial Neural Network (ANN) for compensating for the disadvantage of SEM. ANN is an analysis method inspired by the human nervous system that enables learning by example from representative data describing a physical occurrence or a decision process. The ability of ANN to build empirical relationships between independent and dependent variables, as well as extract delicate information and complicated knowledge from representative data sets, is a distinguishing property. ANNs must be trained in many cases. By training, the relationships between independent and dependent variables can be constructed without making any assumptions regarding a research model.

**Reviewer #2:**

***DEAR Author,***

***The manuscript “ASSESSING ACCEPTANCE OF AUGMENTED REALITY IN NURSING EDUCATION’ is quite interesting, but for contribution some sections should clarify for publication.***

***-Page 4, the 2. Theory section, suggest a change subject title to Background or Literature review, it’s would be better.***

As suggested by the reviewer, on page 4, the section title was changed to “Literature Review”.

***- Why focus on human anatomy with AR in nursing education, it’s unclear? Suggest auditors add references to support the issue with human anatomy in nursing education.***

As suggested by the reviewer, on page 5, the following sections have been added.

For example, using the AR application made it easier for students to grasp subjects that they had difficulty comprehending with the other classical methods. At the same time, it was reported that the AR technology provided the skill and experience regarding the practice. The AR technology does not only create rich content but also provides users to actively practice-related topics in addition to transferring the underlying knowledge. The AR technology allows users to drag, transform, enlarge, or reduce images, and change ways of seeing, manipulating, and experimenting, which can favor the development of the simulation process. AR mobile technology allows nursing students to repeat the clinical nursing practices as many times as they like and study, and practice without worry of making errors. It can also enhance learning results by enhancing students' motivation, engagement, and ability, as well as providing students with knowledge persistence.

On page 6:

It was suggested the nursing students who were enrolled in an internal medicine nursing course, download AR applications AsthiAR and SnapLearn on human anatomy through the Google Play Store to their mobile phones and use them as auxiliary resources to make a self-learning environment efficient. None of the students could do their clinical nursing practices internships in 2019 and 2020 because they were postponed and rescheduled to the 2021-2022 academic year due to the COVID 19 pandemic. In this way, it is expected that they will acquire knowledge and practice human anatomy, which is important for both the nursing courses and internships and the negative effects of the Covid-19 pandemic on students’ education lives will be minimized as much as possible.

***-Section 3.3. Sample and Data Only presented “The Research population for this study was nursing students. The data were collected online from 446 nursing students by using simple random sampling between November 2020 and January 2021.” How to conduct AR with nursing class with intervention process is very vague.***

As suggested by the reviewer, on page 12, the following section has been added.

The target population of this study is nursing students. The data was collected between November 2020 and January 2021 online from the nursing students who have enrolled in an internal medicine nursing course. None of the participants could do their clinical nursing practices internships in 2019 and 2020 because they were postponed and rescheduled to the 2021-2022 academic year due to the COVID 19 pandemic. 446 students voluntarily participated in the study. They were informed that the data will be used for only scientific purposes and their personal information will be kept confidential. Of the 446 administered surveys, 27 were excluded due to being improperly completed. The sample includes 310 female and 109 male students, 63 grad and 356 undergrad students, and the average age is 21.

***--Page13~16, the context contribution related to all ANN described, it’s not clear for the reader, and include tables, for example, what’s the ANN1,2,3…….10? if authors want to keep the section, please address more on the context related to all ANN.***

As suggested by the reviewer, on page 11, the following sections have been added.

One disadvantage of using SEM is the possibility of ignoring the unforeseen relationships in the research model because it analyzes through defined relationships. Therefore, in the second stage, the nonlinear relationships between the constructs were tested with an Artificial Neural Network (ANN) for compensating for the disadvantage of SEM. ANN is an analysis method inspired by the human nervous system that enables learning by example from representative data describing a physical occurrence or a decision process. The ability of ANN to build empirical relationships between independent and dependent variables, as well as extract delicate information and complicated knowledge from representative data sets, is a distinguishing property. ANNs must be trained in many cases. By training, the relationships between independent and dependent variables can be constructed without making any assumptions regarding a research model. Using ANN also gives a chance to verify the results of SEM. Since it is difficult to develop a hypothesis according to the ANN method, it is recommended to use the hybrid model instead of solely the ANN method.

On Page 19,

As seen in Table 4 and Table 5, ANN models were trained ten times. By training, the relationships between independent and dependent variables can be constructed without making any assumptions regarding a research model^60^.

On Table 4,

| Training | Model A-BI | | | Model B-UB | | |
| --- | --- | --- | --- | --- | --- | --- |
| Steps | Training  RMSE | Testing  RMSE | R^2^ | Training  RMSE | Testing  RMSE | R^2^ |
| ANN1 | 0,0951 | 0,0844 | 0,9910 | 0,0972 | 0,0887 | 0,9906 |
| ANN2 | 0,0926 | 0,0942 | 0,9914 | 0,0978 | 0,0881 | 0,9904 |
| ANN3 | 0,0929 | 0,0964 | 0,9914 | 0,0941 | 0,0983 | 0,9911 |
| ANN4 | 0,0875 | 0,1034 | 0,9923 | 0,0975 | 0,0892 | 0,9905 |
| ANN5 | 0,0943 | 0,0896 | 0,9911 | 0,0906 | 0,1062 | 0,9918 |
| ANN6 | 0,0968 | 0,0859 | 0,9906 | 0,0932 | 0,1002 | 0,9913 |
| ANN7 | 0,0957 | 0,0841 | 0,9908 | 0,1023 | 0,0769 | 0,9895 |
| ANN8 | 0,0914 | 0,0937 | 0,9916 | 0,0967 | 0,1030 | 0,9906 |
| ANN9 | 0,0908 | 0,0957 | 0,9917 | 0,1007 | 0,0817 | 0,9899 |
| ANN10 | 0,1061 | 0,1095 | 0,9887 | 0,0988 | 0,0904 | 0,9902 |
| Average  RMSE | 0,094 | 0,094 | 0,991 | 0,097 | 0,092 | 0,991 |
| Standard Deviation | 0,005 | 0,008 | 0,001 | 0,003 | 0,009 | 0,001 |

On Table 5

| Training | Model A-BI | | | | | Model B-UB | | |
| --- | --- | --- | --- | --- | --- | --- | --- | --- |
| Steps | PE | EE | FC | SI | PV | BI | U | HM |
| ANN1 | 0,496 | 0,071 | 0,096 | 0,107 | 0,229 | 0,495 | 0,199 | 0,305 |
| ANN2 | 0,522 | 0,086 | 0,061 | 0,066 | 0,265 | 0,411 | 0,255 | 0,334 |
| ANN3 | 0,500 | 0,041 | 0,059 | 0,170 | 0,231 | 0,480 | 0,265 | 0,255 |
| ANN4 | 0,529 | 0,095 | 0,061 | 0,074 | 0,242 | 0,505 | 0,186 | 0,310 |
| ANN5 | 0,547 | 0,041 | 0,049 | 0,118 | 0,245 | 0,437 | 0,207 | 0,356 |
| ANN6 | 0,480 | 0,066 | 0,083 | 0,112 | 0,259 | 0,492 | 0,218 | 0,290 |
| ANN7 | 0,505 | 0,059 | 0,081 | 0,123 | 0,232 | 0,472 | 0,174 | 0,354 |
| ANN8 | 0,531 | 0,078 | 0,073 | 0,099 | 0,220 | 0,434 | 0,231 | 0,334 |
| ANN9 | 0,556 | 0,054 | 0,061 | 0,085 | 0,245 | 0,516 | 0,212 | 0,272 |
| ANN10 | 0,555 | 0,053 | 0,069 | 0,092 | 0,231 | 0,486 | 0,183 | 0,331 |
| Average Loadings | 0,522 | 0,064 | 0,069 | 0,105 | 0,240 | 0,473 | 0,213 | 0,314 |
| Normalized Importance of Constructs | 100% | 12,7% | 13,5% | 20,6% | 46,5% | 100,0% | 45,5% | 67,1% |

***--The context data of “Table 3. The Research Model Evaluation Results” do not match Figure 2. The Research Model Path Analysis, please carefully clarify. So the section needs rewording based on the revised data.***

As suggested by the reviewer, Table 3 was checked. Corrections highlighted in green have been made.

**Table 3. The Research Model Evaluation Results**

|  | Β | R^2^ | f^2^ | Q^2^ | t- Statistics | P | Hypothesis |
| --- | --- | --- | --- | --- | --- | --- | --- |
| BI |  |  |  | 0.420 |  |  |  |
| HM |  |  |  | 0.375 |  |  |  |
| U |  |  |  | 0.499 |  |  |  |
| PE -> BI | 0.463 | 0.642 | 0.372 |  | 10.679 | 0.000^***^ | H_1_ supported |
| EE -> BI | 0.098 |  | 0,014 |  | 2.523 | 0.012^**^ | H_2_ supported |
| FC -> BI | 0.103 |  | 0.019 |  | 2.852 | 0.004^**^ | H_3_ supported |
| SI -> BI | 0.118 |  | 0.019 |  | 3.048 | 0.002^**^ | H_4_ supported |
| A -> BI | 0.013 |  | -0.003 |  | 0.300 | 0.764 | H_5_ not supported |
| PV -> BI | 0.242 |  | 0.080 |  | 5.390 | 0.000^***^ | H_6_ supported |
| BI -> UB | 0.462 | 0.702 | 0.519 |  | 8.876 | 0.000^***^ | H_7_ supported |
| U -> UB | 0.188 |  | 0.042 |  | 7.656 | 0.000^***^ | H_8_ supported |
| HM -> UB | 0.277 |  | 0.088 |  | 4.909 | 0.000^***^ | H_9_ supported |
| U -> HM -> UB | 0.200 |  |  |  | 5.045 | 0.000^***^ | H_10_ supported |

On Page 21:

As seen in Table 3, the constructs having positive and significant relationships with BI construct and supported hypotheses are as follows: PE (β= 0.463, t= 10.68, p= 0.000) H_1_, EE (β= 0.0098, t= 2.52, p = 0.012) H_2,_ FC (β= 0.103, t= 2.85, p = 0.004) H_3_, SI (β= 0.118, t= 3.05, p= 0.002) H_4_, PV (β= 0.242, t= 5.39, p= 0.000) H_6_. There was no significant relationship found between A and BI (β= 0.013, t= 0.3, p= 0.764) and H_5_ was not supported.

…..

….

As seen in Table 3, the constructs having positive and significant relationships with UB factor and supported hypotheses are as follows: BI (β= 0.462, t= 8.87, p= 0.000) H_7_, U (β= 0.188, t= 7.65, p= 0.000) H_8_, HM (β= 0.277, t= 4.90, p= 0.000) H_9_.

***- Figure 3. Model A and Figure 4. Model B, they are so vague, please address more.***

As suggested by the reviewer, on page 19, the following sections have been added, and Fig 3 and 4 have been enlarged.

In Fig 1, two sub-models were generated for the ANN test in the context of the research model. As seen in Fig 3, Model A has five neurons in the input layer. Each neuron is represented by independent constructs that are PE, EE, FC, SI, and PV. BI, the dependent construct has been represented by one neuron in the output layer. As seen in Fig 4, Model B has three neurons in the input layer. Each neuron is represented by independent constructs that are BI, U, and HM. UB, a dependent construct, has been represented by one neuron in the output layer.

As seen in Table 4 and Table 5, ANN models were trained ten times. By training, as seen in Fig 3. Model A and Fig 4. Model B, the relationships between independent and dependent variables can be constructed without making any assumptions regarding a research model.

**Fig 3. Model A**

**Fig 4. Model B**

***-Based on the incorrect data, please reword Discussion and Conclusion section.***

As suggested by the reviewer, corrections have been made. Since the corrections did not change the results of the hypothesis, the discussion and conclusion parts were checked and it was not necessary to edit them.
