# Supplementary material for: Assessing acceptance of augmented reality in nursing education
Source: PLoS One. 2022 Feb 17;17(2):e0263937. doi: 10.1371/journal.pone.0263937 (PMC8853491; doi:10.1371/journal.pone.0263937)
Supplement: Attachment — Submitted filename: Response to ReviewersV02.docx [file pone.0263937.s003.docx]

Dear Editor,

We appreciate you and the reviewers for taking the time to read our work and provide important feedback. Your important and informative feedback has resulted in possible changes in the current version. We attentively considered the comments and did our best to address each one. We hope that the manuscript, following rigorous edits, meets your high expectations. If there are any further constructive remarks, the writers would appreciate them.

The point-by-point replies are provided below. All changes have been indicated in green in the paper. (**The reviewer’s** comments are in ***italics***)

**Reviewer #2**:

Dear reviewer,

We appreciate you for taking your time to read our work and provide important feedback. Your important and informative feedback has resulted in possible changes in the current version. We attentively considered your comments and did our best to address each one. We hope that the manuscript, following rigorous edits, meets your high expectations. If there are any further constructive remarks, the writers would appreciate them.

The point-by-point replies are provided below. All changes have been indicated in green in the paper.

Best regards

***--The context data of “Table 3. The Research Model Evaluation Results” do not match Figure 2. The Research Model Path Analysis, some data incorrect, please carefully clarify.***

Dear reviwer we have checked many times. To make able 3 more understandable, we have added the "Path Coefficients" and "Specific Indirect Effect (Mediator Effect)" heads.

In Fig 1. the Research Model, we added H10 to the mediator pathways.

Table 3 and Figure 2 data overlap. We checked both, the analyzes and the data by performing a new bootstrapping analysis. As you can see, we have also shared the SmartPLS screenshots, our results are correct. And the results of the previous and new bootstrapping analyzes are similar and the significance levels and hypothesis results overlap.

We would like to point out that we have only shared the results that are hypothesized in the Table 3.

**Fig 1. Research Model**

**Table 3. The Research Model Evaluation Results**

|  | Β | R^2^ | f^2^ | Q^2^ | t- Statistics | P | Hypothesis |
| --- | --- | --- | --- | --- | --- | --- | --- |
| BI |  |  |  | 0.420 |  |  |  |
| HM |  |  |  | 0.375 |  |  |  |
| U |  |  |  | 0.499 |  |  |  |
| Path Coefficients | | | | | | | |
| PE -> BI | 0.463 | 0.642 | 0.372 |  | 10.679 | 0.000^***^ | H_1_ supported |
| EE -> BI | 0.098 |  | 0,014 |  | 2.523 | 0.012^**^ | H_2_ supported |
| FC -> BI | 0.103 |  | 0.019 |  | 2.852 | 0.004^**^ | H_3_ supported |
| SI -> BI | 0.118 |  | 0.019 |  | 3.048 | 0.002^**^ | H_4_ supported |
| A -> BI | 0.013 |  | -0.003 |  | 0.300 | 0.764 | H_5_ not supported |
| PV -> BI | 0.242 |  | 0.080 |  | 5.390 | 0.000^***^ | H_6_ supported |
| BI -> UB | 0.462 | 0.702 | 0.519 |  | 8.876 | 0.000^***^ | H_7_ supported |
| U -> UB | 0.188 |  | 0.042 |  | 7.656 | 0.000^***^ | H_8_ supported |
| HM -> UB | 0.277 |  | 0.088 |  | 4.909 | 0.000^***^ | H_9_ supported |
| Specific Indirect Effect (Mediator Effect) | | | | | | | |
| U -> HM -> UB | 0.200 |  |  |  | 5.045 | 0.000^***^ | H_10_ supported |

Note. PE: performance expectancy; EE: effort expectancy; FC: facilitating conditions; SI: social influence; PV: perceived value; A: anxiety; BI: behavioral intention; U: usefulness; HM: hedonic motivation; UB: use behavior. * 0.10; ** 0.05; *** 0.000 at significance level

*-* ***Figure 3. Model A and Figure 4. Model B they are so vague; please address more.***

As suggested by the reviewer, on page 19 (lines 394-4079), the following sections have been added.

ANN works as a feed-forward and backpropagation, multi-layer perceptron consisting of three layers as input, hidden, and output^76^. It is the modeling of the biological neurons in the brain and the synaptic link established between each other by a computer program. In the learning process, neurons form networks by connecting to each other in various ways. These networks have the ability to learn, memorize and reveal the relationship between variables.

In Fig 1. Research model, two sub-models were generated for the ANN test in the context of the research model. As seen in Fig 3, Model A has five neurons in the input layer. Each neuron is represented by independent constructs that are performance expectancy, effort expectancy, facilitating conditions, social influence, and perceived value. Behavioral intention, the dependent construct has been represented by one neuron in the output layer. Behavioral intention, as seen in Fig 4, Model B has three neurons in the input layer. Each neuron is represented by independent constructs that are Behavioral intention, usefulness, and hedonic motivation. Use behavior, a dependent construct, has been represented by one neuron in the output layer. As seen in Fig 3 and Fig 4, Bias, H(1:1), H(1:2)… are the hidden layer of ANN.

***- the context contribution is related to all ANN described. Please address more on*** ***the context related to the Discussion and Conclusion section.***

As suggested by the reviewer, the following sections have been added to the Discussion and Conclusion.

The SEM and ANN modeling utilized in this study aids in understanding the aspects that drive academic use of AR technology. According to the SEM and ANN findings, as seen in Table 3 and Table 5,….

As seen in the results of SEM and ANN in Table 3 and Table 5,…

The results of this study differ from previous research results^45^-^46^-^47^-^48^. Even though using AR technology creates anxiety; both the expected benefit from its use and its acceptance are high. Also, using AR applications motivate students.

As seen in Table 3 and Table 5, the results show that the most significant factor affecting UB is BI….

*Conclusion Section*

The environment of higher education has evolved dramatically, and internet technologies have played a critical role. With the advent of mobile technologies, students and teachers may now produce their own material and interact with other users; when utilized appropriately, these elements have enormous potential to improve the self-learning experience. Despite the promise for self-learning tools, such as AR technology, to assist the self-learning process, their use in virtual classrooms has not made major gains….

AR applications are mostly developed for self-learning. They can also be developed to improve distant online learning methods. Besides enabling students to practice individually, AR technology also has the potential to create virtual classrooms that multiple users can attend and interact simultaneously. Allowing different users to work and practice on a shared case would certainly differentiate AR technology from other conventional online learning applications. Such a feature would provide convenience not only for clinical nursing practices but also for all health education fields.

*-****The authors used artificial neural networks to determine the non-linear relationships between constructs. Please address more in the Discussion and Conclusion section.***

As suggested by the reviewer, the following section has been added to the Discussion (Lines 492-498)

Furthermore, Table 3 and Table 4 are checked, the R^2^ of the ANN models are much greater than the R^2^ of the PLS-SEM study. This suggests that the variations of BI, and UB to utilize in this study are better explained by the ANN architectures. We believe that the higher R^2^ values obtained from ANN research are connected to the deep ANN architecture’s capability for deep learning and capturing non-linear correlations between components. Researchers are thus encouraged to carefully watch and address the non-linearity issue using a multi-stage data analysis with deep learning.

**Reviewer #3:** Dear reviewer,

We appreciate you for taking your time to read our work and provide important feedback. Your important and informative feedback has resulted in possible changes in the current version. We attentively considered your comments and did our best to address each one. We hope that the manuscript, following rigorous edits, meets your high expectations. If there are any further constructive remarks, the writers would appreciate them.

The point-by-point replies are provided below. All changes have been indicated in green in the paper.

Best regards

***(1) in the second paragraph of Introduction, mentioned that online learning can provide learning resources, and then emphasized “The nursing students were advised to download Augmented Reality (AR) applications and use them as an auxiliary source while studying clinical nursing practices”, the logic was not clear. Compared to traditional online learning resources, what are the special aspects of augmented reality learning resources that are not discussed clearly. (78-81 line)***

As suggested by the reviewer, the following section has been added (Lines 78-83)

The nursing students were advised to download the self-learning Augmented Reality (AR) applications and use them as an auxiliary source while studying clinical nursing practices. AR technology allows the students to study virtual organs, vessels, muscles, and bones. Students can rotate any part of the body 360 degrees and examine them in detail. It offers students the opportunity of gaining experience in clinical practices with virtual intervention to virtual wounds created on the real-like human body^6^. This way, students have the chance to do clinical practices many times over and over^7^.

***(2) in the fourth paragraph of introduction, the necessity of this research is not clearly discussed. What is the difference between the characteristics of AR learning resources and traditional online learning materials? Why does nursing education need AR mobile learning resources, and what defects are made up?***

***Second, the part of Literature review.
In the literature review of AR technology, mentioned the affordance and strength of the AR technology. However, the relationship between these advantages of AR technology and nursing education is not highlighted, that is, whether there are some difficulties in the process of teaching knowledge in nursing education, and AR technology can provide solutions to these difficulties, and explain the necessity of applying AR technology to nursing education.***

As suggested by the reviewer, the following sections have been added (Lines 93-96; 121-125)

On the other hand, as a self-learning method, AR technology differs from others. Unlike common e-learning tools, it offers more than just a way of transferring knowledge. With AR technology, students do not just passively receive knowledge but also actively practice it.

It also provides virtual cases, such as a wound on the human body, so nursing students gain experience in clinical practices^6^. This allows nursing students to repeat the clinical nursing practices as many times as they like without the worry of making a mistake. Azuma and his colleagues^8^ define AR technology as an enabling technology.

***Third, the part of Methods.
The methods part introduces the situation of participants and the questionnaire used, but it lacks how the Internal Medicine Nursing Course was carried out during the epidemic, how the learning materials supported by augmented reality technology were designed, and how students used augmented reality materials to carry out learning. It is suggested to add.***

As suggested by the reviewer, the following section has been added (Lines 160-163)

With the AR technology, the students could examine the human body as a whole and each part in detail. And also, virtual wounds could be created. Students were informed that they could the technology on real human bodies, even on their bodies.

***Lastly, the part of the conclusion.
The conclusion and introduction are repeated. For example, lines 488-490 and 87-88 of the introduction repeat "The Council of Higher Education has recommended to all universities that 40% of the courses Opened after the pandemic should be carried out online ". But this example doesn't seem to be directly related to augmented reality and the need for AR in nursing education.***

As suggested by the reviewer, the following sections have been added (Lines 500-505; 513--519)

The environment of higher education has evolved dramatically, and internet technologies have played a critical role. With the advent of mobile technologies, students and teachers may now produce their own material and interact with other users; when utilized appropriately, these elements have enormous potential to improve the self-learning experience. Despite the promise for self-learning tools, such as AR technology, to assist the self-learning process, their use in virtual classrooms has not made major gains.

AR applications are mostly developed for self-learning. They can also be developed to improve distant online learning methods. Besides enabling students to practice individually, AR technology also has the potential to create virtual classrooms that multiple users can attend and interact simultaneously. Allowing different users to work and practice on a shared case would certainly differentiate AR technology from other conventional online learning applications. Such a feature would provide convenience not only for clinical nursing practices but also for all health education fields.
